# On the development and potential influential aspects of prospective science teachers’ competencies for the use of explainer videos for learning in physics education

**DOI:** 10.3389/fpsyg.2026.1681116

**Published:** 2026-04-10

**Authors:** Michelle Hermann, Markus Wilhelm, Dorothee Brovelli

**Affiliations:** Institute of STEM and Sustainability Education, University of Teacher Education Lucerne, Lucerne, Switzerland

**Keywords:** explainer videos, mixed methods, physics education, professional knowledge, professional vision, prospective science teachers

## Abstract

**Introduction:**

Studies point to the potential of explainer videos in science education as a part of STEM-education but at the same time criticize their heterogeneous quality. As explainer videos can only be effective as digital educational resources if both – the quality and the embedding – are adequate, teachers need to be able to evaluate the quality aspects of explainer videos and embed them accordingly in their lessons to potentially enhance learning outcomes. The present study therefore aims to investigate the extent to which science teachers can draw on their competencies for the use of explainer videos from participatory platforms such as YouTube for their teaching.

**Methods:**

The study utilizes a mixed methods approach to investigate the development of (prospective) science teachers’ competencies for formal learning in physics education (*N* = 229). To this end, the (prospective) teachers’ professional vision of learning support features of two explainer videos about concepts for electrical circuits at the lower secondary level is assessed. A proprietary instrument is employed to record and analyze content-specific professional knowledge and subprocesses of professional vision as situated skills related to criteria-based selection and effective integration of explainer videos into learning processes related to concepts for electrical circuits.

**Results:**

Prospective teachers’ professional vision of learning support features is limited but increases with the years of study (F (3, 198) = 11.12, *p* < 0.001). A path model reveals that the level of training in the present sample significantly predicts both the professional vision and the topic-specific PCK. The results further show a lack of correlation between topic-specific PCK and professional vision of learning support features in the explainer videos. Neither the TPACK self-concept nor the educational attitudes towards digital media influence the professional vision on a significant level.

**Discussion:**

The results indicate that prospective teachers are unable to draw on their topic-specific PCK about the electrical circuit for an adequate selection and effective embedding of explainer videos in teaching and learning. This points to the need to strengthen media-related training for prospective science teachers in their subject-specific training with a focus on the selection and integration of explainer videos.

## 1 Introduction

Information and communication technologies (ICT) are quite often used in STEM disciplines such as mathematics and sciences at secondary level (Fraillon, 2025). Whether ICT such as digital media are beneficial for technology-enhanced learning in STEM disciplines such as mathematics and science, depends largely on the ability of teachers to use media in a educationally meaningful way and thus corresponding teacher training (Hillmayr et al., 2020). Accordingly, teacher training for the use of digital media is of crucial importance, with particular emphasis being placed on subject-specific training (e.g., Angeli and Valanides, 2009). This applies in particular to digital media such as videos from participatory platforms such as YouTube, whose distribution has been increasing for years. (e.g., Bitkom Research, 2024; Kross et al., 2021). A more detailed analysis shows that explainer videos play a central role as digital tools in formal and informal learning (Rat für Kulturelle Bildung e. V., 2019). Therefore, explainer videos are increasingly being considered in general and subject-specific educational research. The studies shed light on both the quality of explainer videos as educational media (e.g., Kulgemeyer, 2018; Findeisen et al., 2019) and their use in the classroom (e.g., Lo, 2019; Vonschallen et al., 2024). These studies illustrate also the two-fold challenge that teachers face when using explainer videos in the classroom: Firstly, given the heterogeneous quality of the explainer videos available on participatory platforms such as YouTube (Kulgemeyer, 2018), teachers must use their professional knowledge to select high-quality explainer videos and, secondly, integrate them adequately into their lessons. In line with Kulgemeyer (2018), Vonschallen et al. (2024) point to the inconsistent study situation, which does not allow a clear conclusion to be drawn as to whether explainer videos are more effective for learning than text-based learning materials. In their comparative study Vonschallen et al. (2024) were able to confirm the potential learning support effect of explainer videos compared to text-based learning materials. However, their results also indicate that learning success depends heavily on the individual explainer video selected, as some videos may be inappropriate or misleading (ibid.). This is supported by Schorn’s (2022) interdisciplinary analysis. She points out that, in addition to subject-specific aspects, it has to be considered that explainer videos might have persuasive goals and often pursue commercial interests by generating clicks and likes. These may lead in some cases to an interest-driven presentation of content that can lead to distortions of factual knowledge, whereby the media presentation in explainer videos creates a sense of credibility due to storytelling techniques and an informal communication style (ibid.). In the most unfavourable case, explainer videos can even create illusions of understanding among learners (Kulgemeyer and Wittwer, 2022; see also 2.5.2), which teachers must counteract through criteria-based selection and adequate embedding of explainer videos. The relevance of further research in this field is underlined by the latest OECD Teaching and Learning International Study (TALIS) conducted in 2018 which showed that lower secondary teachers in OECD countries reported lower levels of self-efficacy in teaching using ICT compared to other aspects such as classroom management (OECD, 2019). One possible explanation for the low levels of self-efficacy is a lack of adequate teacher training (e.g., Fernández-Batanero et al., 2022).

The present study aims to investigate prospective science teachers’ competencies for the use of explainer videos for formal learning in physics education A possible way to assess teachers’ ability to integrate digital media is to consider their professional vision with its subprocesses as situation-specific skills, including the decisions that are crucial for media integration as part of the planning of a specific lesson (e.g., König et al., 2024). A proprietary instrument is used to record and analyze content-specific professional knowledge and situated skills related to criteria-based selection and effective integration of explainer videos into learning processes related to concepts for electrical circuits. Using two explainer videos with concepts for electrical circuits for lower secondary school students as stimuli, we investigate the professional vision of learning support features, and the dispositions potentially associated with them, that are necessary for the effective use of explainer videos. In doing so, we aim to gain insights into subject-related educational aspects which potentially need to be emphasized in teacher training in order to enable science teachers to use explainer videos in a way that is effective for learning.

## 2 Theoretical background

### 2.1 Competencies for the use of digital media

Research on technology-enhanced teaching with a focus on integration of ICT including digital media use has gained increased importance in recent years (Scheiter, 2021). The widely referenced Framework for the Digital Competence of Educators (DigCompEdu) which describes professional competencies on a general non-subject-specific level highlights the relevance of the selection of and teaching and learning with digital media by prominently mentioning corresponding competences (Redecker, 2017):

*Competence “Selecting digital resources” within realm “2 Digital Resources”* (Redecker, 2017): *To identify, assess and select digital resources to support and enhance teaching and learning. To consider the specific learning objective, context, pedagogical approach, and learner group, when selecting digital resources and planning their use*.

*Competence “Teaching” within realm “3 Teaching and Learning”* (Redecker, 2017): *To plan for and implement digital devices and resources in the teaching process, so as to enhance the effectiveness of teaching interventions. To appropriately manage and orchestrate digital teaching interventions. To experiment with and develop new formats and pedagogical methods for instruction.*

In order to further refine the description of competencies for the use of digital media in science education, Thoms et al. (2022) have presented a framework specific for science education that is intended to serve as a ‘structuring aid for an interdisciplinary and integrative approach’ (ibid., 5), focusing on working methods and practices such as documentation or data processing in science education, on which the development of subject-specific competencies can be based, which are the subject of the present study.

For the present study, we conceptualize the above-mentioned competences relating to the selection, planning, and implementation of digital media as situation-specific skills for integrating ICT into lesson planning as presented by König et al. (2024). Based on their scoping review of empirical studies on the integration of ICT in teachers’ lesson plans König et al. (2024) conceptualize context-specific cognitive as well as affective-motivational prerequisites for the integration of ICT. With relation to the PID model of competence-as-a-continuum (Blömeke et al., 2015a) they are seen as dispositions that are functionally responsive to situations and demands for lesson planning with a focus on the integration of ICT as a situation-specific skill (König et al., 2024). According to the model, situation-specific abilities include the perception of learning-relevant situations, their interpretation and the decision for an adequate action in the situation based on its analysis (Blömeke et al., 2015a). These situation-specific abilities can already be found in the concept of ‘professional vision’ developed by Goodwin (1994) as a knowledge-based process. Accordingly, professional vision can focus on different aspects of teaching and learning processes and thus enable the identification of relevant areas of knowledge. Van Es and Sherin (2008) transferred the concept of professional vision to mathematics teaching by defining the two facets of noticing (knowledge-based selective perception) and knowledge-based reasoning (theory-based interpretation) of learning-relevant events in the classroom as sub-processes of professional vision. In the context of a teacher training program with video clubs, they qualitatively assessed the ability to perceive and interpret students’ mathematical thinking. They were able to show that the ability to perceive professionally can be considered as an indicator of teachers’ flexibility (ibid.). In recent years, professional vision has also been researched in other subject areas, with video-based studies showing that professional vision is a knowledge-based domain-specific construct and that PCK and professional vision are positively associated, both for elementary science teaching (Meschede et al., 2017) and for physics teaching (Wöhlke and Höttecke, 2022). In their work, Wöhlke and Höttecke (2022) also point out the importance of valid measurement of professional vision, which can be achieved by ensuring that the aspects to be perceived are not obvious and that professional vision is focused on subject-specific rather than general educational aspects.

When capturing facets of professional competencies, objective proximal approaches are superior to subjective self-assessments or objective distal approaches (e.g., via characteristics that are not related to teaching, such as the level of training of teachers) (Kunter and Klusmann, 2010). Tried and tested objective approaches with proximal indicators include vignette tests (e.g., Oser et al., 2010; Brovelli et al., 2013) or surveys of perception skills using tools such as the Observer (Seidel and Prenzel, 2007; Jahn et al., 2011). Such approaches are able to capture competencies from various perspectives by presenting teaching situations in texts or video clips in a comparatively authentic and situational way, in their complexity and contextuality (see Oser et al., 2010). Following König et al. (2024), planning of a lesson is not only based on professional knowledge on ICT of teachers but also bound to contexts such as knowledge on the subject-specific education of a concept, learners and learning objectives. We therefore developed a proprietary instrument with a specific planning situation around the two selected explainer videos to investigate the situation-specific skills of media selection and integration sensu Redecker (2017) as described in section 3.2.1 The conceptualization of professional knowledge as a disposition for these situation-specific skills is presented in the following section.

Within the frame of competence-as-a-continuum in the context of the integration of ICT, the popular Technological Pedagogical Content Knowledge (TPACK) model (Mishra and Koehler, 2006) specifies the professional knowledge for the integration of ICT in teaching and learning as a cognitive disposition. The widely used theoretical framework based on Shulmans’ conceptualization (Shulman, 1987) of pedagogical content knowledge (PCK) adds the technology-related component of media use and describes seven knowledge domains as dispositions that teachers need to effectively use digital media in the classroom including the context of media use (Koehler and Mishra, 2009). The model emphasizes the importance of ‘Teachers with broad and deep disciplinary knowledge, including subject-specific knowledge, awareness of common alternative conceptions, and multiple levels of scientific models, [who] can provide rich learning opportunities for their students’ (Khourey-Bowers and Fenk, 2009). Following the idea of the framework, teachers with a high level of TPACK need to have a combination of high levels of technological knowledge and pedagogical knowledge as well as content-specific knowledge (Zinger et al., 2017). Factors such as the availability of resources and infrastructure as well as teachers’ beliefs influence the development of TPACK (ibid.). Since its development, the model has repeatedly been criticized due to the lack of empirical delimitation of the different areas of knowledge (e.g., Chai et al., 2016; Willermark, 2018). On the one hand, the often missing distinction between TPACK as knowledge vs. TPACK as competence (Willermark, 2018) and, on the other hand, the limitation of capturing the constructs by means of self-declarations make it difficult to empirically separate the individual knowledge dimensions of the TPACK-framework (see also Section 2.3). Among other aspects, Wohlfart and Wagner (2022) criticize in addition that the simplicity of the framework inadequately reflects the complexity of teaching with digital media and further advocate for an operationalization of the individual knowledge dimensions.

In their review on existing systematic reviews and meta-analyses, Schmid et al. (2024) further identify a systematic lack of clarity when it comes to the meaning of knowledge within the TPACK model, for instance with regard to the question, whether the knowledge domains of TPACK are integrative or transformative, and propose an alignment with general research on PCK and teachers’ professional knowledge which is concerned with similar questions – for instance regarding the refined consensus model RCM (Carlson et al., 2019) with relevance for research on STEM teachers’ PCK (Mientus et al., 2022). Further Schmid et al. (2024) suggest to clearer specify the knowledge that is measured in a specific research project and to derive an appropriate operationalization. In their RCM, which specifies PCK for teaching science and is thus also of relevance for teaching science with digital media, Carlson et al. (2019) define the mechanisms und sources for science teachers’ PCK and differentiate for instance between personal PCK which is manifested in enacted PCK (ePCK) during a teaching cycle which consists of the three phases plan, teach and reflect. The ability to plan for teaching a specific topic is seen as part of ePCK (ibid.). As the cognitive ‘PID-skills’ related to situations teachers need to deal with in class – ‘perception’, ‘interpretation’ and ‘decision making’ (Blömeke, 2024) – also skills for lesson planning are situation-specific skills within the PID model of competence-as-a-continuum (König et al., 2024).

### 2.2 Topic-specific PCK as cognitive disposition for the integration of explainer videos

Following the suggestions of Schmid et al. (2024), the presented study specifies the professional knowledge investigated and focuses on a specific teaching situation with explainer videos as digital media in physics education as a part of STEM education and, with this conceptualization, also takes up the concern for a subject-specific consideration of the use of digital media, as postulated with ‘digital PCK’ in the ProfiLe-P project (Gramzow et al., 2013). The aim of the teaching in focus in this study is to increase lower secondary students’ understanding of electrical circuits, as proposed, for example, in the Next Generation Science Standards framework as an extension of the understanding of energy storage and transmission (National Research Council, 2012). An understanding of electrical circuits requires an understanding of the key variables in a circuit: voltage and current (Burde and Wilhelm, 2020). Earlier research on teaching central conceptions of electric circuits revealed a series of common students’ misconceptions such as the idea that current is consumed by a light bulb or a lack of differentiation between the concepts of voltage and current, which is why Ivanjek et al. (2021) recommend to specifically promote the understanding of voltage as the potential difference between two points. To help students develop this understanding, various analogies – i.e. the water pressure difference as an analogy for voltage as a potential difference – have become established in the classroom (e.g., Burde and Wilhelm, 2020). The importance of analogies for developing an understanding of electrical circuits, with physical processes like electron movement that elude direct perception, reveals the potential added value of explainer videos, which, as digital media with their audiovisual representations, can promote the development of coherent knowledge structures relating to electrical circuits and their key variables (Härtig et al., 2021). This also illustrates the importance of explainer videos for science education as part of STEM, where the study of models and analogies— which can be seen in the visual representations in explainer videos— plays a central role in building conceptual understanding (Coll et al., 2005). Consequently, the study uses Schödl’s 2017 instrument (see Section 3.2.2) to assess topic-specific PCK of learners’ conceptions of electrical circuits, the significance of which in the context of the use of digital media has been demonstrated by Schubatzky et al. (2023). However, the effective use of explainer videos by teachers requires not only an adequate scientific understanding of the concepts to be taught (CK), but also topic-specific PCK of electrical circuits, which includes, in particular, common learners’ conceptions on the electric circuit. Given the relevance of an adequate scientific understanding and the strong positive relationship between CK and PCK shown for models of professional competence in physics (Sorge et al., 2017; Schiering et al., 2023), it would also be reasonable to examine CK in relation to electrical circuits. However, due to the focus on PCK and situation-specific skills and for reasons of test economy, this aspect was not included in this mixed methods study.

### 2.3 TPACK self-concepts and educational attitudes toward digital media as affective-motivational dispositions

According to the PID model, affective-motivational facets are dispositions for situated abilities alongside cognitive facets. In connection with the effective use of digital media in learning, it is therefore necessary to consider not only topic-specific PCK as a facet of subject-specific professional knowledge as a cognitive disposition but also attitudes toward digital media and self-concepts as affective-motivational dispositions. When it comes to the implementation of digital technology, affective-motivational dispositions – for example a person’s self-confidence in accomplishing a specific technology-related task or their perception of the usefulness of a technology – are significant predictors (Farjon et al., 2019). In their empirical study on the use of technology in mathematics education, Backfisch et al. (2020) were able to show the importance of motivational aspects for teachers, such as the perceived usefulness of a technology for effective technology integration. This significance was confirmed in connection with the assessment of TPACK self-efficacy in a subsequent interdisciplinary study focusing on the development of digital literacy with the use of technology (Backfisch et al., 2021), although no statements could be made about the quality of the use of technology. Scherer et al. (2018) were also able to show that educational attitudes toward digital media are more closely related to content-related areas of TPACK than general attitudes, which are more closely related to technology-related areas of TPACK. Self-reported measures are common for the measure of TPACK self-concepts and are implemented often, as TPACK self-concepts influence the acceptance of the digital media use in classrooms (Scherer et al., 2018). Among others, Drummond and Sweeney (2017) point out the lack of validity of survey instruments for self-reported TPACK facets due to a lack of objectivity, which must be supplemented by additional objective metrics if actual knowledge is to be measured. Fabian et al. (2024) confirmed this criticism in their empirical study on the relationship between test-based and self-reported TPACK and the use of technology in mathematics teaching, which also showed the limited informational value of self-reported measurements of TPACK.

### 2.4 A theoretical framework for competencies for the use of explainer videos for learning in physics education

**Figure 1:**
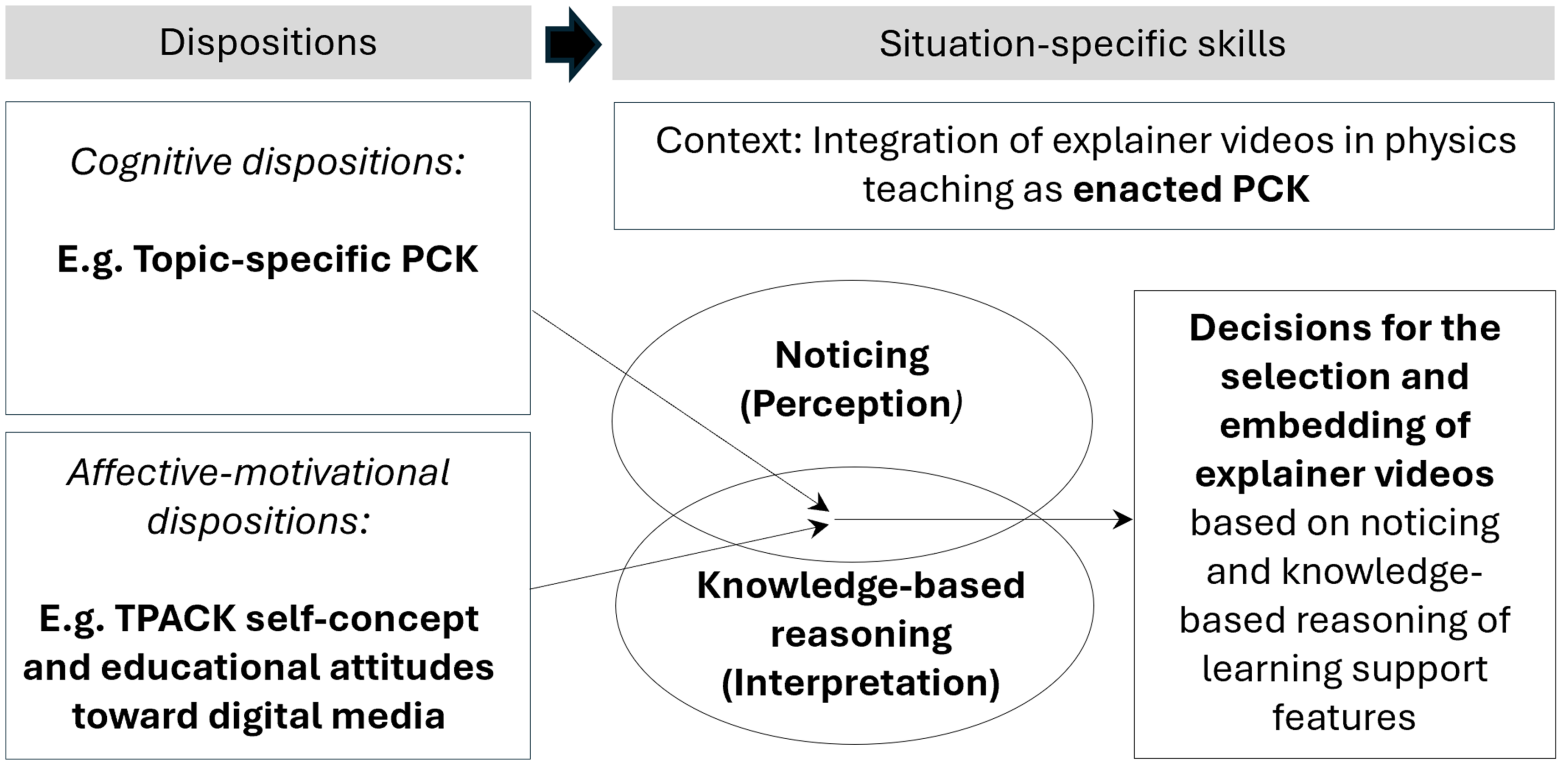
Competencies for the use of explainer videos for learning in physics education (own representation based on König et al., 2024; Blömeke et al., 2015a; Blömeke et al., 2015b).

The theoretical framework underlying this study on the development and potential influential aspects of prospective science teachers’ competencies for the use of explainer videos for learning in physics education is derived based on the heuristic of König et al. (2024), which summarizes based on the PID model (Blömeke et al., 2015a) the conceptualizations encountered in the scoping review on ICT integration in teachers’ lesson plans (König et al., 2024). The heuristic in Figure 1 illustrates our understanding in line with Blömeke (2024), that different models of professional vision (Goodwin, 1994; van Es and Sherin, 2008; Seidel and Stürmer, 2014) are contextualized as situation-specific skills in the PID model by Blömeke et al. (2015a). The concepts and their overlaps are relevant for the operationalization of the situation-specific skills in the presented study. As a consensus on the conceptualization of these frameworks is still lacking – especially when it comes to the aspect of decision making (see also Bastian et al., 2022) – we positioned this third facet in a separate frame.

In addition to teachers’ different competencies for the use of digital media, the characteristics of digital media also influence whether the use of media supports learning. The following section therefore deals with the quality of explainer videos as prominent examples of digital learning media.

### 2.5 Importance and quality of explainer videos as educational media to support learning

#### 2.5.1 Explainer videos as digital learning media

Explainer videos from participatory platforms such as YouTube are prominent representatives among digital learning media which are also in use in formal and informal education. These short films aim to explain in an educational way ‘how to do something or how something works’ (Wolf and Kratzer, 2015) and intend to elucidate abstract concepts. Explainer videos are often not created by trained experts and are therefore based on ‘subjective educational theories’ and the instructional experiences of the creators themselves from school or explanatory formats from television (Rummler and Wolf, 2012). This is reflected in the wide variance in the educational preparation and media design of the school-related explainer videos offered on YouTube (Cwielong and Kommer, 2020) and leads to excessive differences in explanation quality (Kulgemeyer and Peters, 2016) which is countered by a lack of quality control with regard to the explanations in explainer videos on participatory platforms (ibid.). Theoretical considerations on the quality of explainer videos have been presented from both a media and a science education perspective, with subject-independent cognitive psychology findings often being used (e.g., Findeisen et al., 2019; Siegel and Hensch, 2021). Among other aspects, the labeling of relevant content or personalization through direct address while reducing redundant or unclear design elements (e.g., loud background music) are considered conducive to learning (Mayer and Moreno, 2003). From a subject-specific teaching perspective, the quality of the explanation itself is relevant. Among other aspects, the explanation should take into account the ideas and interests of the recipients and support the development of mental models of the presented concepts with appropriate animations and representations (Kulgemeyer, 2018). Kulgemeyer (2018) validated these proposed quality criteria as learning support features in an empirical study (see section 2.5.2).

In terms of design and address, explainer videos are often more informal than educational or documentary films and can be both fact-oriented and entertaining (Rummler and Wolf, 2012). In formal education, young learners consider explainer videos to be important for revising and deepening content, as well as for doing homework and preparing for exams (Rat für Kulturelle Bildung e. V., 2019). In addition, explainer videos also play a central role in science communication and in marketing companies and products (Cwielong and Kommer, 2020), but this is not relevant to the present study, as it focuses on the professional competencies of prospective teachers in formal education in physics. In their empirical study on supporting aspects for learning in the production of instructional videos within massive open online courses based on 6.9 million video watching sessions, Guo et al. (2014) were able to show that the typical viewing time for instructional videos is usually 6 min and that longer videos are often stopped halfway through. The authors therefore recommend watching videos with a playing time of less than 6 min, which was considered when selecting videos for this study.

Rummler and Wolf (2012) point out that learners do not judge the quality of explainer videos on the basis of formal criteria, but intuitively. When explainer videos are embedded on YouTube, learners can interact with the providers and other users via comments and select videos based on likes, views, or comments (Cwielong and Kommer, 2020). However, Kulgemeyer and Peters (2016) empirically showed for explainer videos with content from the fields of astronomy and mechanics in physics that a large number of content-related comments is a better initial indication of the quality of the explainer video than the much more obvious likes or views, which are promoted by the platform as an indication of quality. In our view, these findings underline the importance of the professional selection and embedding of explainer videos against the backdrop of their popularity. However, to our knowledge, little is known about how teachers select explainer videos.

#### 2.5.2 Learning support features in explainer videos

From a general theoretical perspective, explainer videos can support the acquisition of knowledge and skills more effectively than written texts by combining spoken text and visual representations (Mayer and Logan, 2021). From a perspective of science teaching, learning support features such as aspects of cognitive activation and content structuring have proven to be central to the development of performance (Kunter and Voss, 2011). These learning support features can also be found in explainer videos on science topics, among which the quality of the explanations given is naturally of central importance. While interdisciplinary instruments for describing learning support features in explainer videos focus on general educational aspects such as the inclusion of learners’ prior knowledge, the correct representation of learning content and interactivity (Siegel and Hensch, 2021; Findeisen et al., 2019; Ring and Brahm, 2024), the systematic review by Aumann et al. (2023) on student-generated explainer videos and animations in science classes emphasizes the importance of depicting dynamic processes through representations in the form of animations as specific features of explainer videos for science education as part of STEM. This applies in particular to explanations for processes that are not visible to the human eye. To characterize explainer videos in physics education, Kulgemeyer (2018) developed a widely acclaimed rubric consisting of seven factors for the explanatory quality of explainer videos (ibid. 9), which were then validated on the basis of two self-produced videos of different quality. Also this paper examines explainer videos from a subject-specific educational perspective. In order to record the professional vision of learning support features in explainer videos on variables in electrical circuits, where visualizations with models and analogies are of crucial importance (see also 2.2), the rubric of Kulgemeyer (2018) was further developed.

Schwanewedel et al. (2018) emphasize, that media decisions must be the subject of subject-specific educational considerations (ibid., 21) and thus points to the consideration of such subject-specific quality criteria. Nevertheless, also multimedia design plays a role in assessing the learning support effect of explainer videos (Bruckermann et al., 2020). Among other aspects, empirical research based on the cognitive theory of multimedia learning by Mayer and Logan (2021) could confirm modality effects, according to which information presented can be learned better when texts in videos are read aloud rather than written out (Mayer and Moreno, 2003). At the same time, in view of the cognitive load theory by Sweller and Chandler (1994), it must be assumed that explainer videos that are overburdened with content and design elements can lead to an increased extrinsic load on the working memory and in this case the explainer videos cannot develop their learning effect. In order to use explainer videos in a way that is effective for learning, teachers must therefore also take media aspects into account. Several studies have already been conducted on the quality and learning effectiveness of explainer videos in science teaching (e.g., Kulgemeyer, 2018). Among other findings, Kulgemeyer and Wittwer (2022) point out in their experimental study, which used explainer videos developed in-house according to validated criteria, that explainer videos containing misconceptions can lead to an illusion of understanding – defined as a mistaken belief that an explanation was adequate and resulted in understanding – among learners, which the authors consider to be a relevant problem given the heterogeneous quality of explanations in explainer videos. However, in addition to these studies on the learning effectiveness of explainer videos, to our knowledge little is known about how prospective and current teachers use explainer videos in a way that is effective for learning.

### 2.6 Aim and research questions

Regarding the reported low levels of self-efficacy in teaching using ICT (OECD, 2019) and the heterogenous quality of widely used explainer videos (Kulgemeyer, 2018), the aim of the presented study is to shed light on prospective teachers’ perception of explainer videos and their skills in relation to the appropriate integration of explainer videos into science lessons including potential influential aspects such as their TPACK self-concept and their educational attitudes towards digital media.

Since explainer videos have been used more frequently for some time now (Rat für Kulturelle Bildung e. V., 2019), and have been used in particular during the coronavirus pandemic to explain concepts in distance learning (Voss and Wittwer, 2020), it could be assumed that teachers already have experience with them and have therefore built up competencies in using this digital medium. However, the findings on the impact of previous experience with technology use in science education by Vogelsang et al. (2019) suggest a more nuanced view: only university use, but not school use, appears to have a positive influence on factors associated with increased competencies, such as attitudes toward digital media and self-efficacy expectations. Accordingly, the question remains open as to whether and how previous experience influences the increase in competencies of prospective teachers in the use of digital media. Research has not yet addressed prospective teachers’ professional vision of learning support features in explainer videos as a specific category within digital media. In their study with complex text vignettes on PCK in the field of science education, Brovelli et al. (2013) showed, that prospective teachers only perceive contradictions and violations to a limited extent. Furthermore, the study by Steffensky et al. (2015) found that the prospective teachers were hardly aware of violations of features that support learning, such as cognitive activation and structuring. To assess whether the professional vision of learning support features in explainer videos follows these patterns, the following question is investigated:

(1) How does prospective teachers’ professional vision of the (lack of) implementation of educational principles in explainer videos develop at various stages of teacher training?

Together with earlier findings from expertise research (e.g., Berliner, 2001), according to which more experienced teachers have better perceptual abilities than novices, the following hypothesis suggests itself:

*Hypothesis1:* Prospective teachers’ professional vision of learning support features is limited but is expanded during teacher training.

According to the PID model of competence-as-continuum (Blömeke et al., 2015a), subject-specific PCK can be regarded as a disposition for the situational ability of professional vision, which Meschede et al. (2017) have already been able to show in the context of science education. However, it is unclear whether PCK can also be transferred in the context of the competencies considered in this study. To assess whether this relation can be found for professional knowledge on the electric circuit in physics education and other dispositions, the following question is investigated:

(2) How is prospective teachers’ professional vision of the (lack of) implementation of educational principles related to their topic-specific PCK, their educational attitudes towards digital media and their TPACK self-concept?

Based on research results of Meschede et al. (2017) and (Scherer et al., 2018) the following hypothesis regarding research question 2 will be tested:

*Hypothesis 2:* Topic-specific PCK, educational attitudes towards digital media and TPACK self-concept have an effect on professional vision in the context of the considered competencies.

## 3 Materials and methods

### 3.1 Study description and design

The presented study aims to record and analyze prospective teachers’ processes of professional vision as teaching competencies by simulating a planning situation for science lessons at lower secondary level. As part of the assessment of situational skills, a standardized measurement tool was developed consisting of explainer videos as stimuli and a coding manual for evaluating participants’ responses. We assume that the two explainer videos have a similar function in the assessment of professional vision as scripted videos do in other surveys (e.g., Piwowar et al., 2018). The selection and characterization of the two explainer videos is based on a rubric for the subject-specific educational quality of explainer videos (see Section 3.2.3).

The study took place in various courses for prospective science teachers at lower secondary level at six Swiss universities of teacher education. The data were recorded from May 2022 to June 2023. Participation in the study was voluntary, and participants had the option to withdraw from the study at any time, even after signing the declaration of consent. The sample consisted of 198 prospective science teachers from years of study (see Table 1). In addition, we can draw on data from 31 active science teachers (29% female) who are *M* = 38.65 (*SD* = 13.00) years old.

**Table 1:** Gender and age of participants by year of study.

Year of study	*n*	Age M (*SD*)	Proportion of female participants
1st	60	24.12 (6.64)	50.00%
2nd	37	224.08 (4.53)	43.24%
3rd	56	25.57 (3.87)	39.29%
4th and 5th	44	25.27 (2.79)	56.82%

As a planning and not a teaching situation was simulated, the participants were allowed to watch the two videos several times, and the designated overall testing time of 90 min was not technically limited, resulting in an effective average test time of 66.21 min (*n* = 191; *SD* = 30.73), whereby the 7 participants who had interrupted the survey for organizational reasons were excluded from the determination of the average test time The responses of the study participants were included in the evaluation as an exceptionally long test time due to a prolonged interruption of the test by individual participants was not a criterion for exclusion.

### 3.2 Measures and materials

#### 3.2.1 Recording of professional vision

**Figure 2:**
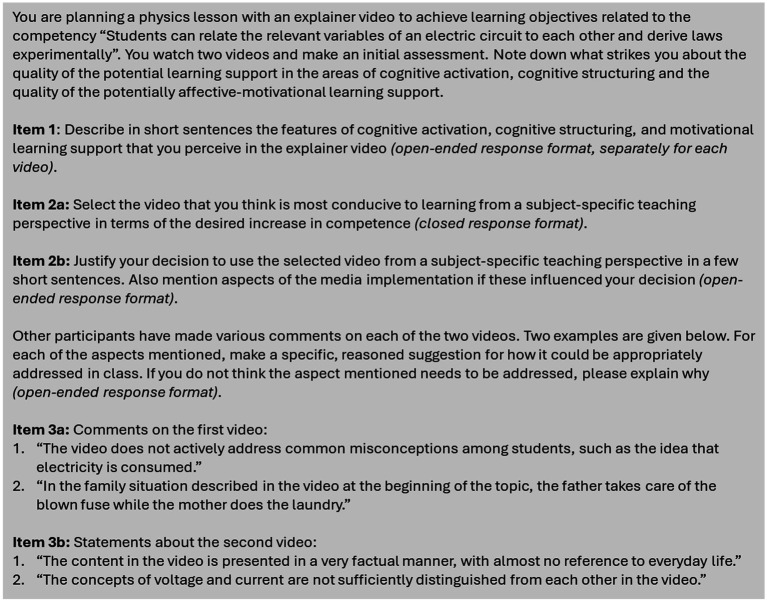
Overview of the items for the recording of professional vision in the context of lesson planning with explainer videos.

The main part of the online survey instrument (implemented in Lime Survey Version 6.14) for this cross-sectional survey consists of three items with an open response format. In the first part we collected demographic and background data such as age, gender, semester and teaching experiences. The participants were then asked to perceive audiovisual information in the selected explainer videos and interpret it based on their professional knowledge as prospective teachers in a similar way that vignette studies using video sequences do (Seidel and Thiel, 2017). To ensure an objective proximal approach, a realistic planning scenario was first provided (see Figure 2). Secondly, to guarantee that the participants’ perceptions are focused on subject-specific teaching, the quality dimensions underlying the characterization of the explainer videos (see section 3.2.3) were specified as part of the task. The participants watch the two selected explainer videos and note firstly what they perceive as positive or negative learning support features (see Item 1 in Figure 2). In line with the conceptualization of Bastian et al. (2022) we expected our participants to ‘see’ learning support features without already interpreting their perception and have a focus on quality-orientated science education. Secondly, they select one of the two videos and justify their choice from an educational point of view based on their professional competencies (see Items 2a and 2b in Figure 2). Finally, in the third item, they comment on how a shortcoming in the video (see section 3.2.3) can be responded to with a suitable implementation in the classroom. This suggestions for implementation are understood as part of planning activities with explainer videos as ICT sensu König et al. (2024) and allow to take up a demand made by Backfisch et al. (2020), according to which the processes underlying the use of technology should be examined directly. To ensure that even those participants who had overlooked the subject-specific educational shortcomings identified in the explainer videos could demonstrate their decision-making skills, they were confronted with deficits identified by the experts in the characterization of the two explainer videos (see categories 3a and 3b in Figure 2). To ensure that participants who overlooked the subject-specific educational shortcomings in the first part of the survey could not subsequently change their answers from the first part, it was technically impossible to scroll back in the survey. Analogous items were developed for two additional areas of competence (one in biology and one in chemistry). For reasons of test economy, participants only completed the survey for two of the three subjects.

#### 3.2.2 Recording of potential influential aspects on professional vision

In addition, dispositions as potential influencing factors on professional vision are surveyed using scales of standardized instruments with open and closed answering format. These include topic-specific PCK related to the electrical circuit (Schödl, 2017), attitudes towards digital media (Scherer et al., 2018) as well as TPACK self-concepts (Schmid et al., 2020). Also, other potential influencing factors such as age or educational background were recorded.

For a valid measurement in the context of the present study, the assessment of topic-specific PCK on the electric circuit should consider both the topic-specific aspects of competence development in relation to voltage and current, as well as two central facets of PCK (Gramzow et al., 2013) – instruction and teaching strategies as well as learner cognitions. The model of the digital media facet of PCK for physics teaching developed in parallel with this work also takes up several of the aforementioned considerations (Große-Heilmann et al., 2022). The associated survey instrument (ibid.) covers the use of various digital media in physics teaching in its entirety (ibid.). In order to ensure the suitability of the measurement of topic-specific PCK in the context of the present work, we selected Schödl’s (2017) FDW scale, which was validated in the context of the Falko-P study (7 items with open-ended response format, *α* = 0.72). With its theoretical foundation in Shulman’s framework of professional knowledge (Shulman, 1987) and the COACTIV model of professional competence (Baumert et al., 2010), it is compatible with the concept of professional knowledge according to the PID model (Blömeke, 2024). In order not to exceed the total test time of 90 min and to put an emphasis on the prospective teachers’ knowledge on common learners misconceptions’ and knowledge on explaining and representations as central elements of topic-specific PCK, we selected 4 items from the sub-facets “Knowledge about explaining and representing content” (E&R in the original study) and “knowledge about typical student difficulties and errors” (Schk in the original study). The selection also considers the particular importance of explanation quality and the quality of learning support features in explainer videos in explainer videos (Example item [FDPHe_01_i]: ‘[…] Briefly and concisely describe as many fundamentally different ways as possible to counteract this “electricity consumption idea” in the further course of the lesson.’). The open-response items were evaluated using the coding manual of the original study.

To assess prospective teachers’ TPACK self-concepts, we used the short scale from the instrument ‘tpck.xs’ by Schmid et al. (2020) comprising of five items where participants indicated their TPACK self-concept using a five-point response format, where a higher value represents a stronger consent (Sample item [self_tpck2]: ‘I can select digital technologies for my teaching that improve what I teach, how I teach, and what students learn.’, *α* = 0.80).

For the recording of the educational attitudes towards digital media, we use the adapted and translated short scale ‘attitudes towards ICT in education’ of Scherer et al. (2018), comprising of three items. The participants indicated their attitudes towards digital media in education using a six-point response format, where a higher value also here represents a stronger consent and thus a more positive attitude (Sample item [edatt2]: ‘The use of digital media enables me to prepare my lessons better’, *α* = 0.77).

#### 3.2.3 A rubric of quality criteria for explainer videos

As a basis for the characterization of the explainer videos used as stimuli and the generation of the expert norm to compare the answers of the participants with, we developed a rubric with quality criteria which summarizes learning support features with relevance in science education. To ensure a focus on science education, the rubric is based on learning support features which are associated with high-quality teaching (Praetorius and Nehring, 2020), and therefore structured along the principles of cognitive activation and cognitive structuring. Against the background of the long-known relevance of affective-motivational aspects for learning in science education (Glynn et al., 2009), the rubric is supplemented by affective-motivational characteristics.

Based on the parallels between the research areas of technology-enhanced teaching research and teaching and learning in the field of cognitive processing in learning (Scheiter, 2021), characteristics from teaching research were combined with characteristics for effective explainer videos to form a rubric that allows explainer videos to be assed. For the assembly of the quality criteria we combined established quality aspects of science teaching (Heinitz and Nehring, 2020) with quality criteria for explainer videos from existing rubrics with a general educational focus (Siegel and Hensch, 2021), a focus on media education (Findeisen et al., 2019) and physics education (Kulgemeyer, 2018). This resulted in a rubric with twelve quality criteria in three target dimensions which is presented in Table 2.

**Table 2:** Features of potential learning support as criteria for the quality of explainer videos.

Dimension	Learning support features	Description
Potentially cognitively activating features	Implicit integration of learners’ concepts	The video refers to the previous knowledge of the audience by anticipating pre-instructional understanding of learners during the design phase and incorporating them into explanations.
	Explicit addressing of learners’ concepts	The video does not reinforce misconceptions through incorrect presentation (linguistic or pictorial).
	Activating prior knowledge	The video provides cognitive activation by making the learner aware of their learning status through references to related concepts or required prior knowledge.
	Embedding in multiple contexts	In the video, concepts are considered in different situations and from multiple perspectives.
Potentially cognitively structuring features	Encouraging conceptual thinking	The content conveyed in the video is placed in its overarching subject context, thus building logical structures.
	Development of mental models through representations	The relationships between the presented concepts are made clear in the video, and viable models for the presented phenomena are introduced.
	Optimization through clarity of content	The video presents content in a (scientifically) correct and comprehensible way (this also applies to images, text and graphics).
	Limiting to the essentials	The video highlights the essential terms and elements of the scientific concept.
Features that potentially support learning in an affective-motivational manner	Arouse positive feelings	The video arouses fascination and enthusiasm through spectacular phenomena or humorous presentation.
	Demonstrate relevance	The video clarifies the relevance of the subject matter through appropriate contextualization.
	Arouse curiosity	The video stimulates curiosity through the novelty value of its content, thereby increasing interest.
	Enabling identification	Presenters can potentially act as role models and appeal to the emotions of the audience together with the content.

The first target dimension of cognitive activation includes the integration and addressing of students’ concepts, the activation of prior knowledge and embedding of subject-specific concepts in multiple contexts. By specifically incorporating aspects of physics education such as pre-instructional understanding in the form of learner conceptions, it addresses a key criticism raised by Wöhlke and Höttecke (2022), namely that existing instruments for measuring professional vision are often invalid because they focus professional vision on general educational aspects rather than subject-specific ones. The second target dimension of cognitive structuring includes the promotion of conceptual thinking and the development of mental models, as well as optimization through clarity of content and a focus on the essentials. The third target dimension of motivational-affective learning support includes evoking positive feelings, demonstrating relevance, arousing curiosity and enabling identification by addressing the audience directly. This rubric of quality criteria was used to characterize the explainer videos in terms of their potential to support learning from a subject-specific educational perspective. Since the learning effectiveness of the explainer videos used as stimuli was not examined, no direct conclusions can be drawn from the rubric regarding the learning effectiveness of the used videos.

#### 3.2.4 Selection of explainer videos as stimuli

This study examines the use of explainer videos as part of formal education. Accordingly, questions arise in connection with lesson planning activities such as video selection that are similar to those that arise when selecting representations in text and images in school textbooks. Seidel and Thiel (2017) point out that the selection of existing videos as stimuli for the measurement of competencies should be carried out with the help of differentiated criteria based on subject-related educational models. To select the explainer videos used as stimuli, the research team therefore applied criteria commonly used in connection with videos for teaching and learning (Piwowar et al., 2018), such as relevance (e.g., targeting an appropriate level of learners) and realism (e.g., authenticity of the video content). The selected videos address curricular relevant subject-related concepts of electrical circuits with a special focus on representations in the form of analogies which are used to explain the key variables within an electric circuit – voltage and current (Burde and Wilhelm, 2020). To ensure the authenticity of the stimuli, two videos were selected which, based on the research team’s practical experience, can realistically be used to achieve an increase in competence at the corresponding level in the field of electricity according to the Swiss curriculum ‘Lehrplan 21’: ‘Pupils can relate the relevant variables of a simple electrical circuit to each other and derive laws experimentally’ (D-EDK, 2016). From a formal point of view, attention was paid to the comparability of the content of the two selected explainer videos and to a playback time between 5 and 6 min, in order to ensure that the explainer videos are watched to the end (Guo et al., 2014). References regarding the two explainer videos on voltage and current used for the survey can be found in Table 3.

**Table 3:** References regarding the two explainer videos on voltage and current used in the study.

Explainer video	Reference
Explainer video 1	Physik – simpleclub. (2021, 28 August). Strom and Spannung Grundlagen. [Video]. YouTube. The video is no longer publicly available.
Explainer video 2	Gymi Neubi. (2025, 28 December). Spannung und Stromstärke. [Video]. YouTube. https://www.youtube.com/watch?v=zEUC610WyG0.

Six physics education experts participated in the rating of the two explainer videos to identify the characteristics of learning support as a basis for the generation of the later expert norm for evaluating the responses of study participants. Each of the experts rated the two videos in a desktop rating process following a survey with closed (5-point rating scale) and open (text) answer formats. The resulting four to eight characteristics of learning support per explainer video, which were recognized by the experts with at least sufficient reliability, form an adequate basis for the coding manual for evaluating the assessments of the learning support features by later study participants.

### 3.3 Data analysis

The results of the expert rating form the basis of the qualitative content analysis according to Kuckartz and Rädiker (2023) for the coding and evaluation of the open-response items for the recording of the professional vision (see Figure 2). The coding manual’s rubrics were derived from a deductive category system based on the expert rating and thus the predefined features of potential learning support as criteria for the quality of explainer videos (see Table 1).

For statements that suggest noticing, we award one point for mentioning a feature relevant to learning and another point if the expression of the feature is also assessed in line with the experts’ rating. We awarded points according to the same principle for answers that could be assigned to the reasoning sub-process, whereby we distinguished here between interpretation of what was perceived and specific suggestions for embedding it in a specific teaching situation. Here, too, additional points were awarded for a high level of agreement with the experts’ rating. This quantification in MAXQDA Analytics Pro 24 (Release 24.1.0) allowed both the breadth of the answers to be honored, via the number of features recognized, and the depth of the answers, via an assessment of the quality of the judgements. The evaluative coding of the responses on learning support features in the explainer videos according to Kuckartz and Rädiker (2023) leads to points for each answer which we added up to form a total score. Statements without subject-related content were assigned to additional categories that were generated inductively in the course of the analysis. For these statements, no points were awarded due to the lack of subject-related content. Two independent raters coded 15% of the participants responses and achieved a substantial inter-rater agreement (*κ* = 0.64) (Brennan and Prediger, 1981). The responses to the four items on PCK on the electrical circuit were analyzed using the coding manual of the original study in an evaluative content analysis. Here two independent raters coded 20% of the participants responses and achieved a substantial inter-rater agreement substantial inter-rater agreement (*κ* = 0.78) (ibid.). The subsequent quantitative data analysis includes descriptive statistical analyses, group comparisons using analysis of variance (ANOVA) and an analysis of the influences of the participants’ PCK related to the electrical circuit on their professional vision using t-tests and regression analyses.

## 4 Results

The results are presented in two sections which address one of the two research questions each.

*Research question 1: how does prospective teachers’ professional vision of the (lack of) implementation of educational principles in explainer videos develop at various stages of teacher training?*

**Figure 3:**
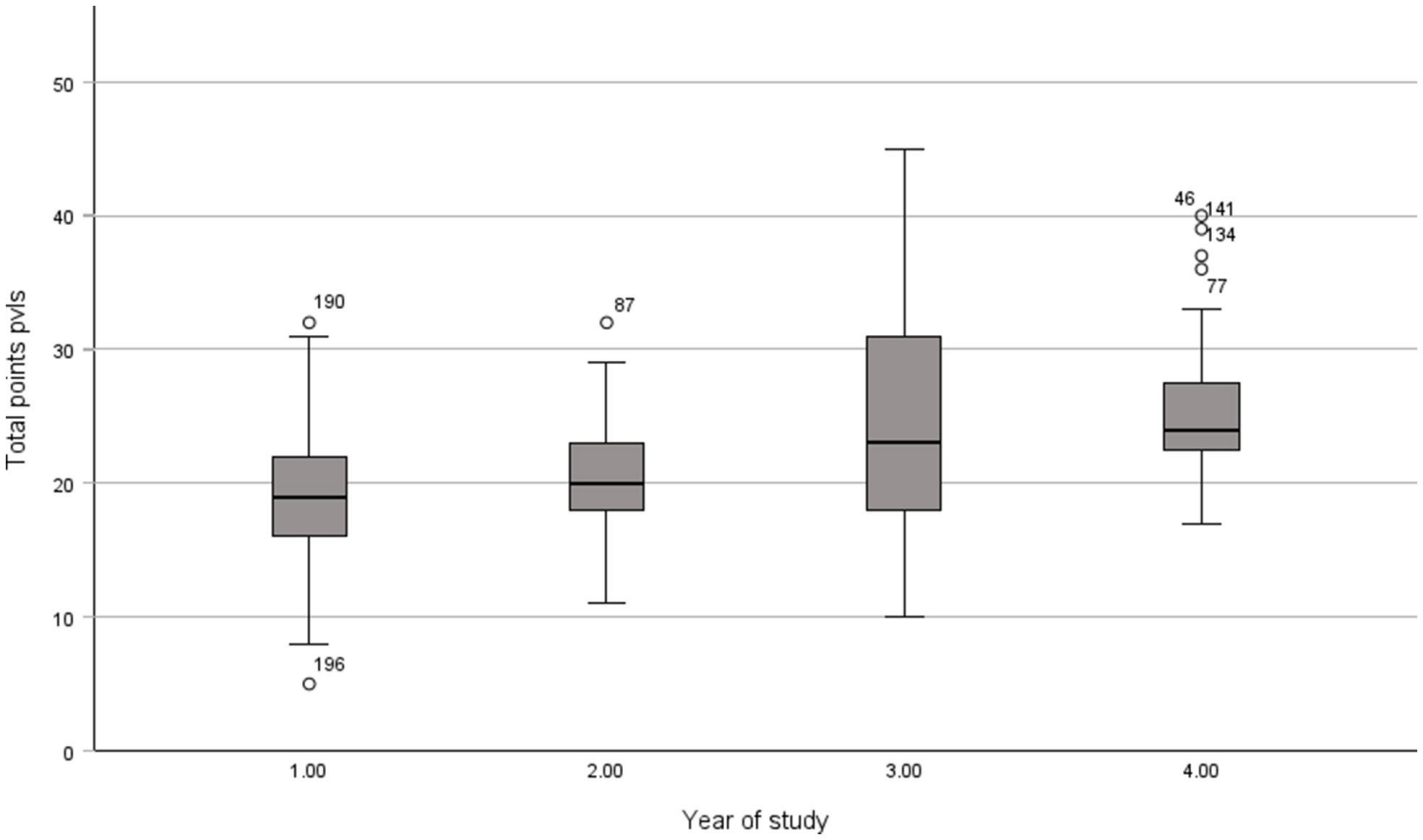
Professional vision of relevant learning support features (pvls) at various stages of teacher training (year of study).

The box plot (Figure 3) shows that the median of points achieved by the participating prospective teachers for professional vision (pvls) increases with the number of years of study. The F-statistics of the Welch-ANOVA (*F* (3, 198) = 11.12, *p* < 0.001) confirm the visual impression of the correlation between total points and level of education. The participants achieved a maximum of 45 points while the vast majority of participants achieved significantly lower scores (*M* = 22.29, *SD* = 6.89). The participants are often unable to notice the expression of learning support features identified by the experts in the expert rating (see section 3.2.4), and their interpretations and proposed embedding (knowledge-based reasoning) differ from those of the experts, which results in not achieving high scores. We can thus accept our hypothesis 1 that prospective teachers’ professional vision of learning support features is limited but is expanded during teacher training.

*Research question 2: how is prospective teachers’ professional vision of the (lack of) implementation of educational principles related to their topic-specific PCK, their attitudes towards digital media and their TPACK self-concept?*

The topic-specific PCK is recorded with the four items with an open-response format on the electrical circuit from the scale validated by Schödl (2017). The median number of points achieved by participants increases from the 2nd year of training for pre-service teachers over the course of their training (*F* (3, 170) = 2.67, *p* = 0.049). In addition to the influence of topic-specific PCK on professional vision in explainer videos, we also examined the influence of educational attitudes towards digital media (edatt). The participants have a clearly positive attitude (*M* = 4.35, *SD* = 1.10) of a comparable magnitude to the respondents in the original study (Scherer et al., 2018). Finally, the participants’ TPACK self-concept was measured using a five-point response format. They have a rather positive TPACK self-concept (*M* = 3.77, *SD* = 0.56) of a magnitude comparable to that of the original study (Schmid et al., 2020).

**Figure 4:**
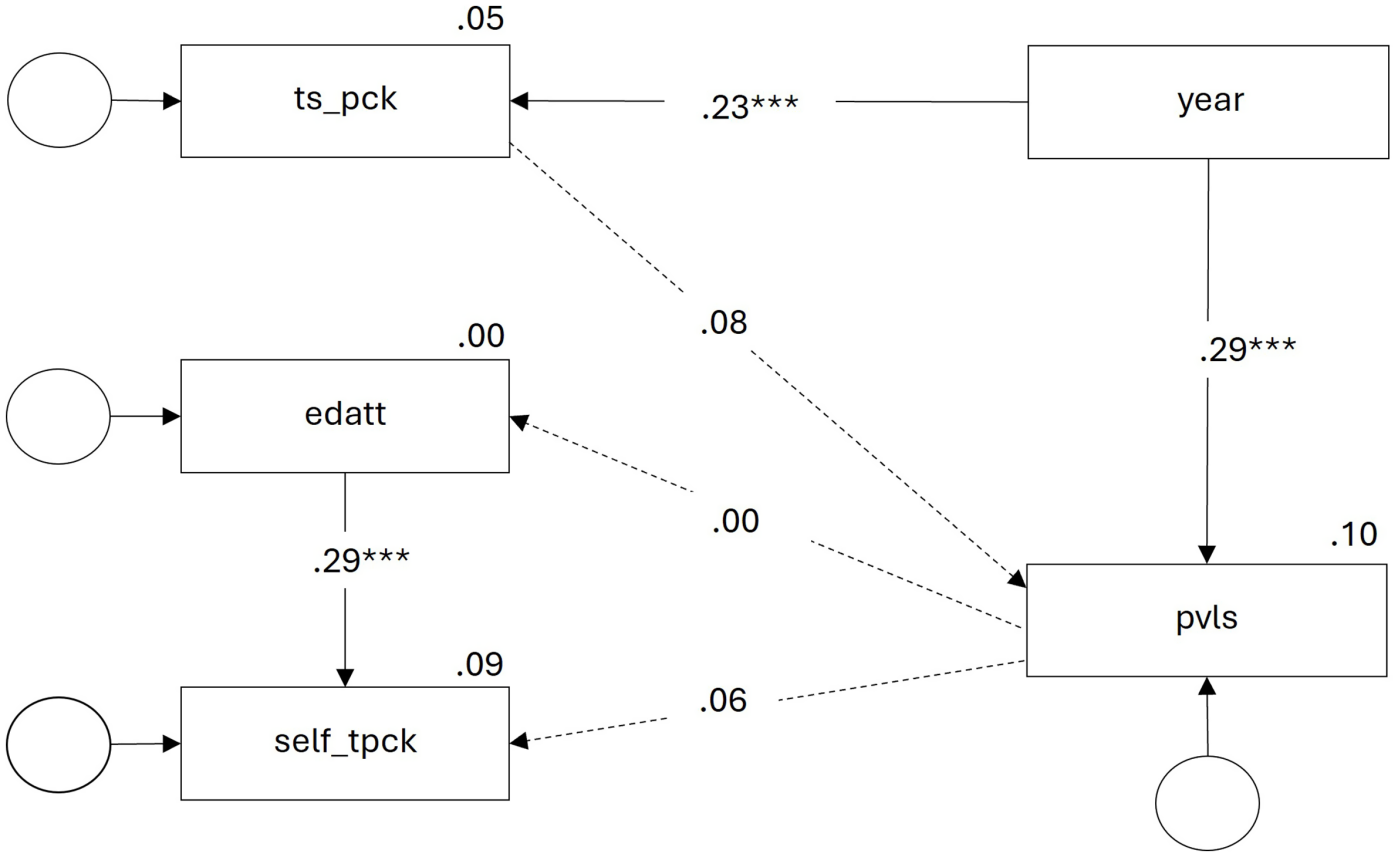
Path model for dispositions for the professional vision of learning support features in physics explainer videos (pvls).

In order to examine the relationships between the professional vision of learning support features and the factors just described, we combined all factors to the path model presented in Figure 4.

On the one hand, the model in Figure 4 shows that the level of training and experience in the present sample (year) significantly predicts both the professional vision of learning support features of the explainer videos (pvls) and the topic-specific PCK (ts_pck). The model reveals no correlation between topic-specific PCK and professional vision of learning support features in the explainer videos. The path coefficients of the two affective-motivational dispositions, educational attitudes towards digital media (edatt) and TPACK self-concept (self_tpck), are insignificant.

The model’s quality criteria in Table 3 indicate a good fit with the data (*χ2* (4) = 4.061, *p* = 0.398).

## 5 Discussion

The presented study looked at the development of professional vision of learning support features in explainer videos on central variables within the electric circuit and potential influencing factors on these situation-specific skills as professional competencies in physics teaching with digital media. The results indicate that prospective science teachers’ topic-specific professional knowledge does not support an adequate selection and effective embedding of explainer videos in teaching and learning. The following section discusses these results and sheds light on limitations.

### 5.1 The development of prospective teachers’ professional vision at various stages of teacher training

Concerning professional vision of learning support features in explainer videos, we assumed that prospective teachers only notice the violation of key principles of science education to a limited extent. Further we assumed that this ability can be developed during teacher training. The majority of the prospective teachers in our study show a lack of perception of learning support features in the two explainer videos and are therefore presumably not able to propose adequate instructional embedding that considers the lack of implementation of principles of science education. This indicates that the pre-service teachers surveyed tend not to have enough competencies to use explainer videos effectively in the classroom.

According to the assessment of the experts, who participated as raters of the two explainer videos used as stimuli in this study, the explainer videos exhibit a number of shortcomings in terms of subject-specific education (Hermann et al., under review). As many of the prospective teachers in the survey did not report on these shortcomings, they seem to be not critical enough when they characterize explainer videos and are therefore not fully exploiting the potential of this digital educational medium. This is in accordance with results from studies on professional vision, which show that prospective teachers, in contrast to experienced teachers, often notice only a small number of aspects of learning support in presented video vignettes (Meschede et al., 2017). Based on the present results, we hypothesize that a significant proportion of participants are subject to an illusion of understanding similar to the one identified by Kulgemeyer and Wittwer (2022) for first-year students of physics when using explainer videos. In line with the research results of Kulgemeyer and Wittwer (2022), who found a comparable illusion of understanding among learners, it is assumed that prospective teachers also need guidance and support when selecting explainer videos for effective teaching.

### 5.2 Relations between prospective teachers’ professional vision and potentially influencing factors

With research question two, we shed light on the influence of different dispositions according to the model of competence-as-a-continuum. Accordingly, topic-specific PCK can be regarded as a disposition for the situation-specific skill of professional vision, which Meschede et al. (2017) and colleagues have already been able to show in the context of science teaching. Our analyses show that students in higher semesters and with more experience have a better professional vision of learning support features in physics explainer videos. However, they are unable to apply their existing topic-specific PCK on teaching concepts of the electrical circuit on the perception and interpretation of learning support features in the explainer videos about the electrical circuit. The results show a lack of correlation between topic-specific PCK and professional vision of learning support features in the explainer videos which suggests that the transition from topic-specific PCK to professional vision of learning support features as a situation-specifc skill for the planning of teaching with digital media does not occur smoothly despite the broad use of explainer videos and experience with it. One possible explanation might be that the participants’ professional vision of learning support features is limited due to the high cognitive load (Sweller and Chandler, 1994) associated with the density of information in the explainer videos. With regard to the theoretical framework developed in accordance with König et al. (2024), it also seems plausible that their limited professional vision of learning support features in the explainer videos prevents them from undertaking the adequate embedding, which is so important for effective learning (Kulgemeyer, 2018; Vonschallen et al., 2024). Against the backdrop of the concern raised by Schorn (2022) about the high persuasive power based on characteristics of explainer videos such as storytelling techniques and informal communication style – which in certain cases may distort the facts they contain – this perception is significant not only from a subject-specific educational perspective, but also from a media teaching perspective.

These results can be read as an indication of the need for additional awareness-raising among pre-service and in-service teachers regarding the quality aspects of explainer videos. The lack of transfer makes it clear that awareness-raising must also include media-specific aspects. Ideally, these approaches are combined with subjects-specific educational aspects that are particularly relevant to science education with explainer videos as part of STEM, such as depicting dynamic processes through representations in the form of animations to build up understanding (Coll et al., 2005), particularly for processes that are not visible to the human eye (Aumann et al., 2023).

### 5.3 Limitations and further directions

The results presented here focus on two explainer videos on the electrical circuit used as stimuli and the specific aspects of physics education that arise from it. In order to gain good access to the field of research, the survey was mainly conducted among prospective teachers who have limited teaching experience. A meta-analysis of *k* = 92 eye-tracking studies by Keskin et al. (2024) demonstrated that the professional vision of pre-service teachers with limited teaching experience differs significantly from that of in-service teachers. According to Keskin et al. (2024), in-service teachers focus their gaze on learners significantly more often during lessons than pre-service teachers, who focus their gaze on instructional material or other objects in the classroom (ibid.). Given the aforementioned background, it is reasonable to conclude that – although the present study addresses explainer videos rather than instructional videos – the findings of the present study cannot be readily transferred to the situation of in-service teachers. A subsequent evaluation of the data collected in this study will examine the open-ended response formats of a sub-sample with regard to these differences. The aim is to gain further insights into how the professional vision of prospective teachers can be better promoted in subject-specific teacher training. To obtain insights into the current situation in the professional educational field and to derive generalizable conclusions, the study would need to be extended to a larger sample of teachers already working in the profession in a next step. Given that the study takes an explicitly educational perspective on explainer videos (see also Table 2), we decided not to collect additional data on CK related to electrical circuits in this mixed methods study for reasons of test economy, despite the empirically proven positive relationship between CK and PCK (Sorge et al., 2017; Schiering et al., 2023). It stands to reason that including CK on electrical circuits in a follow-up study could provide an explanation and should therefore be considered. Due to the ratio of the sample size to the scope of the model, no latent modeling could be performed, which limits the significance of the relations found. To verify the relations, a further survey with a more comprehensive sample would have to be conducted, which would enable latent modeling. The effect observed may also be due to the assumed one-dimensionality of professional vision in the path model. With regard to current research for mathematics teachers’ on the connections between the facets of situated abilities and the associated dispositions (Yang et al., 2021), according to which the connections between professional knowledge and the sub-processes of interpretation and decision-making are stronger than those between the sub-process of noticing (ibid.), more in-depth analyses on the level of the sub-processes of professional vision could provide further clarity. Other promising research perspectives would be to examine the learning effectiveness of the videos used, as this would also provide empirical insights for the development of effective explainer videos for creators of videos, or misconceptions on the electric circuit as a potential influence on the professional vision of learning support features.

## 6 Conclusion

Our study analyzed the competencies of prospective science teachers in the evaluation, selection and integration of explainer videos for physics education on the electric circuit. The results can be read as indications that prospective teachers are not able to fully exploit the potential of explainer videos as educational media in their later professional life, since they do not recognize deficits in the explainer videos and therefore cannot integrate them in a way that is effective for learning. This suggests a need for additional awareness-raising for quality aspects of explainer videos as part of the training of science teachers. We recommend that science teachers’ awareness of quality aspects of explainer videos should be raised as part of their subject-specific training to take account of the heterogeneous quality of explainer videos from participative platforms such as YouTube. This is in line with recommendations of Krey and Rabe (2021) who further suggest that also learners at lower secondary level should be trained to look at the quality of explainer videos and bring the self-production of explainer videos into play as another meaningful learning arrangement.

## Data Availability

The raw data supporting the conclusions of this article will be made available by the authors, without undue reservation.
